# A Calculation Method of Bearing Balls Rotational Vectors Based on Binocular Vision Three-Dimensional Coordinates Measurement

**DOI:** 10.3390/s24196499

**Published:** 2024-10-09

**Authors:** Wenbo Lu, Junpeng Xue, Wei Pu, Hongyang Chen, Kelei Wang, Ran Jia

**Affiliations:** 1 School of Aeronautics and Astronautics, Sichuan University, Chengdu 610065, China; 2022226210008@stu.scu.edu.cn (W.L.); pwei@scu.edu.cn (W.P.); wangkelei_@stu.scu.edu.cn (K.W.); 2023226210008@stu.scu.edu.cn (R.J.); 2 Robotic Satellite Key Laboratory of Sichuan Province, Chengdu 610065, China; 3 Xichang Satellite Launch Center, Xichang 615000, China; chy89728@163.com

**Keywords:** vision measurement, motion model construction, rotational vectors measurement

## Abstract

The rotational speed vectors of the bearing balls affect their service life and running performance. Observing the actual rotational speed of the ball is a prerequisite for revealing its true motion law and conducting sliding behavior simulation analysis. To address the need for accuracy and real-time measurement of spin angular velocity, which is also under high-frequency and high-speed ball motion conditions, a new measurement method of ball rotation vectors based on a binocular vision system is proposed. Firstly, marker points are laid on the balls, and their three-dimensional (3D) coordinates in the camera coordinate system are calculated in real time using the triangulation principle. Secondly, based on the 3D coordinates before and after the movement of the marker point and the trajectory of the ball, the mathematical model of the spin motion of the ball was established. Finally, based on the ball spin motion model, the three-dimensional vision measurement technology was first applied to the measurement of the bearing ball rotation vector through formula derivation, achieving the analysis of bearing ball rolling and sliding characteristics. Experimental results demonstrate that the visual measurement system with the frame rate of 100 FPS (frames per second) yields a measurement error within ±0.2% over a speed range from 5 to 50 RPM (revolutions per minute), and the maximum measurement errors of spin angular velocity and linear velocity are 0.25 °/s and 0.028 mm/s, respectively. The experimental results show that this method has good accuracy and stability in measuring the rotation vector of the ball, providing a reference for bearing balls’ rotational speed monitoring and the analysis of the sliding behavior of bearing balls.

## 1. Introduction

The bearing structure is the core support component of many rotating machines and is widely used in large, heavy-duty equipment such as wind turbines and aircraft engines. Bearing balls are susceptible to heat and slip during high-speed rotational motion, leading to early failure of the bearing well before it reaches its design life [1,2]. In addition, turbines and engines are becoming increasingly integrated and complex in industrial production. Accordingly, mechanical components such as bearings, gears, and couplings are more prone to failures attributed to frequent shocks and vibrations [3]. The rotational speed and direction of the balls have a significant impact on the service life and normal operation of the bearings. Hence, observing the actual rotational speed of the balls can provide accurate and real physical data for the simulation and analysis of the motion law and sliding behavior. Currently, there is a lack of measurement methods for the real data of rotational speed in the field of rotating machinery. Many measurement methods are only based on the observation of the rotational speed of rotating parts, and there is a lack of deep study on the motion parameters related to the revolution and spin of the ball. It is difficult to measure the spin angular velocity of the ball at high speeds. Therefore, it is urgent to carry out research on the measurement of spin angular velocity during the high-speed motion of balls.

At present, many scholars have studied the motion laws and modeling of the rolling ball. Oktaviana investigated the effect of ball bearing pressure at different speed ranges and the effect of ball rotational speed on bearing slip behavior according to the Hirano criterion [4,5]. Gupta developed a complete set of kinetic models to analyze the motion and sliding of the balls considered in the bearing [6]. Chen gave a quasi-static model of a ball bearing, establishing creep-slip and spin ratios to accurately define the relative motion between the balls and the inner and outer raceways [7]. Ostensen modeled the dynamics of ball motion [8]. Imado proposed a hall element method for detecting ball motion in ball bearings [9]. Selvaraj developed a test rig for cylindrical roller bearings to measure the rotational speed of bearing elements under various operating conditions [10]. Wang investigated the dynamic characteristics of ball bearings and gave a method for determining the angular velocity and pitch angle of the balls [11]. In these studies, the motion model of the ball was considered and the motion law of the ball was analyzed; however, the rotation vectors, such as the spin angular velocity of the ball, were not actually measured.

The binocular vision measurement technology has been widely used in the fields of object three-dimensional coordinate measurement and morphology estimation due to its non-contact, low impact from the external environment, and strong anti-interference ability. Ye combined the high-speed rotation image acquisition of low-speed cameras with the association of 3D digital images to search for uncoded targets between two rotating images. The 3D motion parameters obtained through 3D reconstruction and singular value decomposition enable full-field 3D motion and deformation measurement of high-speed rotating objects [12]. Bao introduced rotor speed as a Kalman filter parameter and combined it with prior information on rotating machinery to construct an extended Kalman filter model, achieving dynamic axial clearance measurement of high-speed rotating machinery [13]. Wang decomposed the deformed image and reference image into multi-resolution layers and converted the two-dimensional template into a one-dimensional grayscale value sequence. The correlation coefficient between the two comparison images was calculated in the low-resolution layer, achieving the estimation of the initial values of the translation and rotation speed of the rotating object [14]. Jia proposed a method for calibrating projector parameters in a DCP system with high accuracy, which uses the binocular camera to uniquely determine the coordinates of 3D feature points [15]. Yu proposed an adaptive binocular fringe dynamic projection method that can avoid image saturation by adaptively adjusting the projection intensity and achieving higher accuracy for high dynamic range three-dimensional vision measurement [16]. Xu proposed a contactless measurement method based on vision technology and realized the measurement of the rotation angle of power transformer winding coils [17]. In these studies, the three-dimensional coordinates of the feature points and the rotation angle of the components are measured using visual and image methods; however, the rotation vector of the components is not further measured.

In recent years, binocular vision measurement technology has been applied to the field of measuring the rotational speed of rotating components. Furuno proposed a rotation rate estimation algorithm based on a rotation matrix, which measured the rotation rate and translation speed of the ball [18]. Liao proposed a method of using cameras and marker tracking to measure the instantaneous speed of wind turbine blades, which achieved better measurement accuracy and speed compared to image correlation methods [19]. Wang proposed a rotation speed measurement system based on a low-cost imaging device, which uses structural similarity and two-dimensional correlation algorithms to process the image and complete the measurement of rotor rotation speed [20]. Wang proposed an imaging-based instantaneous speed measurement system and a periodic determination method based on the Chirp-Z transform and parabolic interpolation autocorrelation algorithm, which can effectively measure the rotor revolution speed [21,22,23]. Wang proposed a high-speed stereo vision system that can detect balls and identify marker points from highly underexposed low-resolution images under normal indoor lighting conditions without additional strobes, enabling the measurement of the rotational speed of a golf ball during high-speed motion [24]. Jung proposed a camera-based multi-exposure image acquisition sensor system and ball-tracking algorithm to capture multi-exposure images from two cameras simultaneously using a synchronization controller. In addition, the 2D center point of the ball is extracted from the separated and overlapped images, and the 3D (three-dimensional) coordinate reconstruction of the extracted feature points is performed using the direct linear transformation method to measure the speed of the golf ball [25]. Seo proposed a 3D ball motion estimation system based on an infrared scanning sensor, which can estimate the motion speed and motion angle of a golf ball [26]. Zhang proposed a dual-camera high-speed stereo vision table tennis tracking system in which a computer receives the image coordinates of the ball from the camera and calculates its 3D position in the working frame. The flight trajectory of the ball is estimated and predicted based on the measured position of the ball and the ball motion model. The direction of the velocity of the ball during the motion can be estimated [27]. Xie extracted the features from the left and right images obtained using the binocular camera and a three-dimensional measurement method based on an improved feature-matching algorithm is proposed to improve the measurement accuracy for objects with sparse or weak textures [28]. These studies used visual measurement methods to measure the instantaneous speed and rotational speed of spheres such as ping-pong balls and tennis balls. However, during the movement of these spheres, the surface of these spheres is not blocked by other devices, making it impossible to reproduce the actual application scenarios of bearing structures. In the practical application of bearing structure, the ball inside the bearing will be blocked by various devices, which makes it more difficult to obtain the actual motion image of the ball. Therefore, the rotational vectors of the bearing ball cannot be measured directly by the existing research methods.

Zhang utilized a binocular stereo vision system to track the position of the ball and a frame difference technique as well as a third panning camera to measure the spin angular velocity of the ball, which allowed them to identify the brand of moving table tennis under normal lighting conditions. Then, the 3D attitude of the ball is fitted in the ball-centered coordinate system, and the rotation state of the table tennis was estimated by using a planar fitting method based on the consensus of weighted random samples. The trajectory prediction of the moving table tennis is realized by extended Kalman filtering [29]. Li proposed a high-precision rotation angle measurement method using machine vision to measure the rotation angle, where the coordinates of the points on the calibration map are obtained from the visual image of the calibration map acquired by a CCD camera. The mathematical model of the aberration error of the CCD camera was established while the measurement of the rotation angle of the spot array was realized by using the coordinate rotation measurement equation [30]. Zhong proposed an infrared spectroscopic measurement system based on non-projective stripe vision, in which the designed LVD-FP was completely pasted on the surface of the rotating shaft along the direction of stripe density variation. The streak period density of each imaged LVD-FP intensity changes due to the rotational axis, which realizes the measurement of the rotation angle, the determination of the direction of the rotational axis, and the measurement of the instantaneous angular velocity between two adjacent frames [31]. Kim pasted RGB color patterns on the rotational axis to be tested, and a high-speed camera was used to image the color marker points. By detecting the speed of the RGB color change, the measurement of the rotational speed of the rotary axis is realized [32]. Guo proposed a Lucus–Kanade algorithm based on template matching to accomplish the motion tracking of rotating objects by aligning template images in a video sequence. For the special case of non-planar cylindrical objects, a nonlinear transformation model for rotational tracking is designed to measure the rotational speed of a cylindrical coupling by tracking a rotating object in a video sequence [33].

In summary, when the ball is engaged in high-speed reciprocating motion, it is also engaged in spin motion during revolution, making it impossible to directly measure the spin speed of the ball. The above study used visual measurement methods to measure the rotational speed of moving objects or rotating components but did not accurately measure the spin angular velocity and linear velocity of high-speed moving balls. Thus, aiming at the need for accurate measurement of ball spin vector in mechanical field, and the problem that it is difficult to measure the ball spin angular velocity directly with existing research methods, a mathematical model for calculating the ball spin angular velocity is constructed for the first time. Moreover, a method of measuring bearing ball rotation vector based on marked point vision three-dimensional coordinate measurement is proposed, and the accurate measurement of each bearing ball rotation vector is finally realized, which provides a reference for the monitoring of the bearing running state and analysis of ball sliding behavior.

## 2. Proposed Theories and Methods

### 2.1. Overview of the Principle of Ball Rotational Speed Measurement

**Figure 1:**
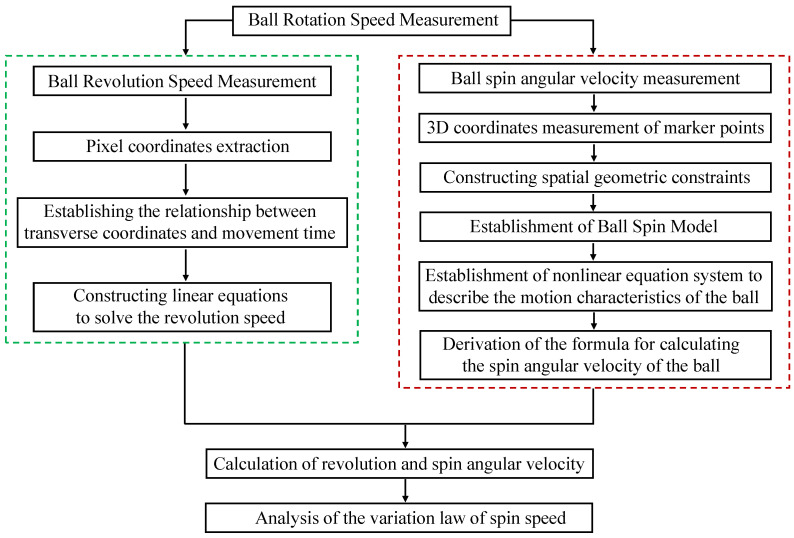
Steps for measuring the rotational vectors of ball bearings.

In order to analyze the sliding behavior of bearing balls, we need to calculate the sliding velocity of the balls. However, the premise for calculating the sliding velocity of the ball is to know the revolution angular velocity and spin angular velocity of the ball. As shown in Figure 1, the measurement of ball rotational speed is divided into two parts: revolution and spin. For spin measurement, we measure the spin angular velocity, spin linear velocity, and sliding velocity of the ball. For revolution measurement, we measure the revolution angular velocity and linear velocity value of the ball.

In the spin measurement part of the ball, a series of marker points with positional configurations are preset on the surface of the ball to be measured first. Secondly, a binocular camera is used to capture the motion process of the ball and analyze the images of adjacent frames to locate and extract the 2D coordinates of the marker points. Then, the 3D coordinates of the marker points can be measured using the triangulation principle. Further, the spatial position of the ball is determined based on the 3D coordinates of the marker points, and the coordinates of the ball center are determined. A spatial circle formed by the movement trajectories of the different marker points is established in the course of the spin movement of the ball. We determined the three kinds of geometric constraints on the marker points made by the ball rotor axis and the ball surface so as to construct a mathematical model of the high-speed movement of the ball and establish a system of nonlinear equations corresponding to the geometric constraints. Finally, the 3D coordinates of the marker points, the coordinates of the ball center, and the radius of the ball are substituted into the constructed nonlinear model for optimization and solution in order to obtain the coordinates of the center of each spatial circle and the radius of the corresponding spatial circle. The spin angle and direction of each marker point per unit time are further calculated to obtain the direction and numerical results of the ball spin angular velocity.

In the revolution measurement part of the ball, the transverse coordinates of the ball in the pixel coordinate system are extracted first. Second, according to the specific correspondence between the transverse coordinates of the ball and the movement time, a linear equation is established to solve for the revolution period of the ball, so as to calculate the revolution speed.

### 2.2. Proposed Ball Spin Measurement Principle

**Figure 2:**
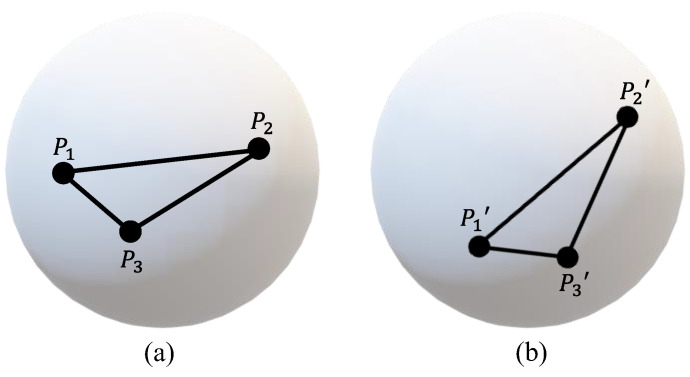
Schematic diagram before and after ball movement: (**a**) Position of the marker points before ball movement. (**b**) Position of the marker points after ball movement.

In the spin measurement of the ball, a series of circular marker points with relative positional configurations were laid on the ball. As shown in Figure 2, the three marker points $P_{1}$, $P_{2}$, and $P_{3}$, are marked on the ball surface by a specific graphene material. This specific graphene material can ensure that the marker points will not be easily worn out during repeated friction. In addition, there is a certain relative positional configuration between the three marker points, which ensures that the selected three marker points can be distinguished at any time. The three marker points before the ball movement are labeled as $P_{1}$, $P_{2}$, and $P_{3}$, and the corresponding three marker points after the ball movement are labeled as $P_{1}^{\prime }$, $P_{2}^{\prime }$, and $P_{3}^{\prime }$. The 3D coordinates of the six points $P_{1}$, $P_{2}$, $P_{3}$, $P_{1}^{\prime }$, $P_{2}^{\prime }$, and $P_{3}^{\prime }$ are calculated using binocular vision principles. Firstly, extract the 2D coordinates of these six points in the image. Then, calculate the 3D coordinates of these six points based on the pre-calibration parameters of binocular vision.

Tests have shown that the spin angular velocity of the ball is much greater than the revolution angular velocity [34]. Thus, the distance of the ball revolution is much smaller than the distance of the marker point’s movement in a short time interval, and the following reasonable assumptions can be made: during a short time of movement, the displacement changes of the marker point on the surface of the ball can be regarded as the displacement change caused by the spin motion of the ball. At this time, the mixed movement of the ball can be regarded as a spin movement only.

**Figure 3:**
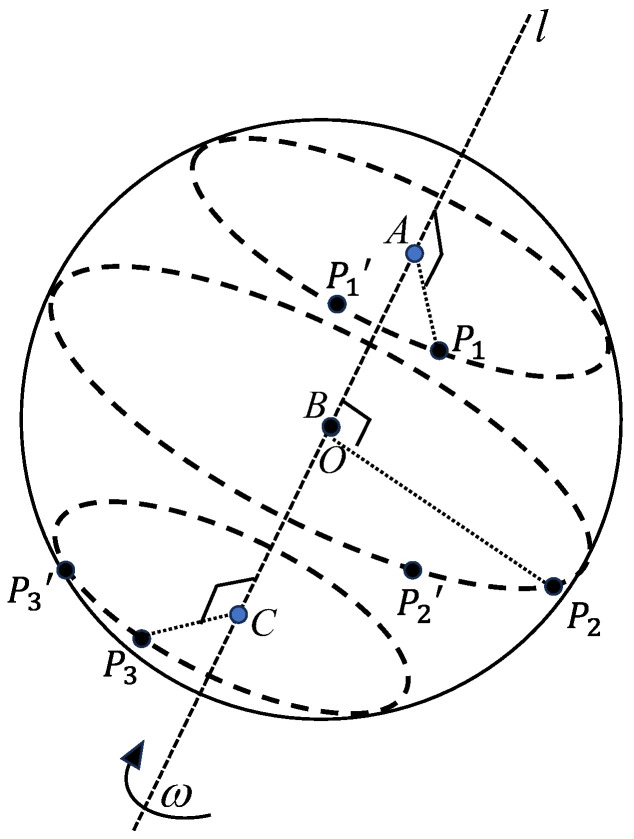
Ball spin motion model.

As shown in Figure 3, for the marker point $P_{1}$, there is only one plane perpendicular to the axis of rotation l and intersecting with l at point A over the line segment $P_{1}$$P_{1}^{\prime }$. This plane intersects with the surface of the ball to form the space circle ⊙A. Then, the points $P_{1}$ and $P_{1}^{\prime }$ are located on the space circle ⊙A centered at the point A and radiused at $AP_{1}$ = ${\mathrm{AP}}_{1}^{\prime }$. For the marker points $P_{2}$, $P_{2}^{\prime }$, and $P_{3}$, $P_{3}^{\prime }$, the space circle ⊙B and circle ⊙C are obtained in the same way.

Let the center of the ball be $O(x_{o},y_{o},z_{o})$, the coordinates of the centers of the space circles be ⊙A, ⊙B, ⊙C be $A(x_{A},y_{A},z_{A})$, $B(x_{B},y_{B},z_{B})$, and $C(x_{C},y_{C},z_{C})$, respectively. The radius of the space circles ⊙A, ⊙B, and ⊙C are given as D, E, and F, respectively. The coordinates of two points on the space circle ⊙A are $P_{1}(x_{P_{1}},y_{P_{1}},z_{P_{1}})$, $P_{1}^{\prime }(x_{P_{1}^{\prime }},y_{P_{1}^{\prime }},z_{P_{1}^{\prime }})$, the coordinates of two points on the space circle ⊙B are $P_{2}(x_{P_{2}},y_{P_{2}},z_{P_{2}})$, $P_{2}^{\prime }(x_{P_{2}^{\prime }},y_{P_{2}^{\prime }},z_{P_{2}^{\prime }})$, and the coordinates of two points on the space circle ⊙C are $P_{3}(x_{P_{3}},y_{P_{3}},z_{P_{3}})$, $P_{3}^{\prime }(x_{P_{3}^{\prime }},y_{P_{3}^{\prime }},z_{P_{3}^{\prime }})$. For this ball spin angular velocity measurement model, the following geometric constraints exist:

(1) Points $P_{1}$, $P_{1}^{\prime }$ lie on the space circle ⊙A, points $P_{2}$, $P_{2}^{\prime }$ lie on the space circle ⊙B, and points $P_{3}$, $P_{3}^{\prime }$ lie on the space circle ⊙C.

(2) The rotary axis l is perpendicular to the plane in which the space circles ⊙A, ⊙B, and ⊙C are located.

(3) The centers of the circle A, B, C, and the center of the ball O share the same line.

Based on the above geometric constraints, a nonlinear equation system can be constructed to describe the motion characteristics of the ball.

(1) Let the spatial circular equations of circles ⊙A, ⊙B, and ⊙C be:

(1) $${\left(x-x_{A}\right)}^{2}+{\left(y-y_{A}\right)}^{2}+{\left(z-z_{A}\right)}^{2}={R_{A}}^{2}$$

(2) $${\left(x-x_{B}\right)}^{2}+{\left(y-y_{B}\right)}^{2}+{\left(z-z_{B}\right)}^{2}={R_{B}}^{2}$$

(3) $${\left(x-x_{C}\right)}^{2}+{\left(y-y_{C}\right)}^{2}+{\left(z-z_{C}\right)}^{2}={R_{C}}^{2}$$

Substituting the 3D coordinates of two points $P_{1}$, $P_{1}^{\prime }$ on the space circle A into Equation (1):

(4) $$\left\{\begin{array}{l} (x_{P_{1}}-x_{A}{)}^{2}+(y_{P_{1}}-y_{A}{)}^{2}+(z_{P_{1}}-z_{A}{)}^{2}={R_{A}}^{2}\\ (x_{P_{1}^{\prime }}-x_{A}{)}^{2}+(y_{P_{1}^{\prime }}-y_{A}{)}^{2}+(z_{P_{1}^{\prime }}-z_{A}{)}^{2}={R_{A}}^{2} \end{array}\right.$$

Similarly, for the space circles ⊙B and ⊙C, substituting the 3D coordinates of $P_{2}$ and $P_{2}^{\prime }$, $P_{3}$, and $P_{3}^{\prime }$ into Equations (2) and (3), respectively:

(5) $$\left\{\begin{array}{l} (x_{P_{2}}-x_{B}{)}^{2}+(y_{P_{2}}-y_{B}{)}^{2}+(z_{P_{2}}-z_{B}{)}^{2}={R_{B}}^{2}\\ (x_{P_{2}^{\prime }}-x_{B}{)}^{2}+(y_{P_{2}^{\prime }}-y_{B}{)}^{2}+(z_{P_{2}^{\prime }}-z_{B}{)}^{2}={R_{B}}^{2}\\ (x_{P_{3}}-x_{C}{)}^{2}+(y_{P_{3}}-y_{C}{)}^{2}+(z_{P_{3}}-z_{C}{)}^{2}={R_{C}}^{2}\\ (x_{P_{3}^{\prime }}-x_{C}{)}^{2}+(y_{P_{3}^{\prime }}-y_{C}{)}^{2}+(z_{P_{3}^{\prime }}-z_{C}{)}^{2}={R_{C}}^{2} \end{array}\right.$$

(2) The rotary axis l is perpendicular to the plane in which the space circles ⊙A, ⊙B, and ⊙C are located, respectively:

(6) $$l⊥P_{1}P_{1}^{\prime }A$$

(7) $$l⊥P_{2}P_{2}^{\prime }B$$

(8) $$l⊥P_{3}P_{3}^{\prime }C$$

According to Equation (6), it is obtained from the line–plane perpendicularity theorem:

(9) $$\left\{\begin{array}{l} \vec{OA}⊥\vec{P_{1}A}\\ \vec{OA}⊥\vec{P_{1}^{\prime }A} \end{array}\right.$$

Substituting the 3D coordinates of the points O, A, $P_{1}$, $P_{1}^{\prime }$ into Equation (9):

(10) $$\left\{\begin{array}{l} (x_{A}-x_{P_{1}})(x_{A}-x_{O})+(y_{A}-y_{P_{1}})(y_{A}-y_{O})+(z_{A}-z_{P_{1}})(z_{A}-z_{O})=0\\ (x_{A}-x_{P_{1}^{\prime }})(x_{A}-x_{O})+(y_{A}-y_{P_{1}^{\prime }})(y_{A}-y_{O})+(z_{A}-z_{P_{1}^{\prime }})(z_{A}-z_{O})=0 \end{array}\right.$$

Similarly, according to Equations (7) and (8):

(11) $$\left\{\begin{array}{c} \left(x_{B}-x_{P_{2}}\right)\left(x_{B}-x_{O}\right)+\left(y_{B}-y_{P_{2}}\right)\left(y_{B}-y_{O}\right)+\left(z_{B}-z_{P_{2}}\right)\left(z_{B}-z_{O}\right)=0\\ \left(x_{B}-x_{P_{2}^{\prime }}\right)\left(x_{B}-x_{O}\right)+\left(y_{B}-y_{P_{2}^{\prime }}\right)\left(y_{B}-y_{O}\right)+\left(z_{B}-z_{P_{2}^{\prime }}\right)\left(z_{B}-z_{O}\right)=0\\ \left(x_{C}-x_{P_{3}}\right)\left(x_{C}-x_{O}\right)+\left(y_{C}-y_{P_{3}}\right)\left(y_{C}-y_{O}\right)+\left(z_{C}-z_{P_{3}}\right)\left(z_{C}-z_{O}\right)=0\\ \left(x_{C}-x_{P_{3}^{\prime }}\right)\left(x_{C}-x_{O}\right)+\left(y_{C}-y_{P_{3}^{\prime }}\right)\left(y_{C}-y_{O}\right)+\left(z_{C}-z_{P_{3}^{\prime }}\right)\left(z_{C}-z_{O}\right)=0 \end{array}\right.$$

(3) The collinear of the four points at the center of the circle, A, B, C, and the center of the ball O can be obtained from the properties of the straight lines in space:

(12) $$\left\{\begin{array}{l} \vec{OA}∥\vec{OB}\\ \vec{OA}∥\vec{OC} \end{array}\right.$$

Substitute the 3D coordinates of points A, B, C, and O into Equation (12):

(13) $$\left\{\begin{array}{l} \frac{x_{A}-x_{O}}{x_{B}-x_{O}}=\frac{y_{A}-y_{O}}{y_{B}-y_{O}}=\frac{z_{A}-z_{O}}{z_{B}-z_{O}}\\ \frac{x_{A}-x_{O}}{x_{C}-x_{O}}=\frac{y_{A}-y_{O}}{y_{C}-y_{O}}=\frac{z_{A}-z_{O}}{z_{C}-z_{O}} \end{array}\right.$$

Organize Equation (13):

(14) $$\left\{\begin{array}{l} (x_{A}-x_{O})(y_{B}-y_{O})-(y_{A}-y_{O})(x_{B}-x_{O})=0\\ (x_{A}-x_{O})(z_{B}-z_{O})-(z_{A}-z_{O})(x_{B}-x_{O})=0\\ (y_{A}-y_{O})(z_{B}-z_{O})-(z_{A}-z_{O})(y_{B}-y_{O})=0\\ (x_{A}-x_{O})(y_{C}-y_{O})-(y_{A}-y_{O})(x_{C}-x_{O})=0\\ (x_{A}-x_{O})(z_{C}-z_{O})-(z_{A}-z_{O})(x_{C}-x_{O})=0\\ (y_{A}-y_{O})(z_{C}-z_{O})-(z_{A}-z_{O})(y_{C}-y_{O})=0 \end{array}\right.$$

In the above equations, the unknowns are the center of the ball $O(x_{o},y_{o},z_{o})$, the center of the circle $A(x_{A},y_{A},z_{A})$, $B(x_{B},y_{B},z_{B})$, $C(x_{C},y_{C},z_{C})$, and the radius of the spatial circle $R_{A}$, $R_{B}$, $R_{C}$—15 unknown parameters in total. If the coordinates $P_{1}(x_{P_{1}},y_{P_{1}},z_{P_{1}})$ and $P_{1}^{\prime }(x_{P_{1}^{\prime }},y_{P_{1}^{\prime }},z_{P_{1}^{\prime }})$, $P_{2}(x_{P_{2}},y_{P_{2}},z_{P_{2}})$ and $P_{2}^{\prime }(x_{P_{2}^{\prime }},y_{P_{2}^{\prime }},z_{P_{2}^{\prime }})$, $P_{3}(x_{P_{3}},y_{P_{3}},z_{P_{3}})$ and $P_{3}^{\prime }(x_{P_{3}^{\prime }},y_{P_{3}^{\prime }},z_{P_{3}^{\prime }})$ of the moments before and after each marker point are known, the above system of nonlinear equations can be solved and optimized.

First of all, after using $P_{1}$, $P_{1}^{\prime }$, $P_{2}$, $P_{2}^{\prime }$, $P_{3}$, $P_{3}^{\prime }$—six points of the 3D coordinates to determine both the center of the ball coordinates $O(x_{o},y_{o},z_{o})$ and the radius of the ball uniquely—and substituting $P_{1}(x_{P_{1}},y_{P_{1}},z_{P_{1}})$, $P_{1}^{\prime }(x_{P_{1}^{\prime }},y_{P_{1}^{\prime }},z_{P_{1}^{\prime }})$, $P_{2}(x_{P_{2}},y_{P_{2}},z_{P_{2}})$, $P_{2}^{\prime }(x_{P_{2}^{\prime }},y_{P_{2}^{\prime }},z_{P_{2}^{\prime }})$, $P_{3}(x_{P_{3}},y_{P_{3}},z_{P_{3}})$, and $P_{3}^{\prime }(x_{P_{3}^{\prime }},y_{P_{3}^{\prime }},z_{P_{3}^{\prime }})$ into the system of nonlinear equations, then, the system of nonlinear equations can be solved and optimized to obtain the coordinates of the center of the circle A, B, C, and the radius of the spatial circle $R_{A}$, $R_{B}$, $R_{C}$, and then the spatial circle ⊙A, ⊙B, and ⊙C with known parameters can be constructed in the space.

The coordinates of the center and radius of each spatial circle are known; therefore, the ball spin direction and the magnitude of the spin angular velocity can be solved. First, it is known that the four points A, B, C, and O are collinear, and let (x,y,z) be any point on the rotation axis l; then, the linear equation of l in space is:

(15) $$\frac{x-x_{A}}{x_{B}-x_{A}}=\frac{y-y_{A}}{y_{B}-y_{A}}=\frac{z-z_{A}}{z_{B}-z_{A}}$$

**Figure 4:**
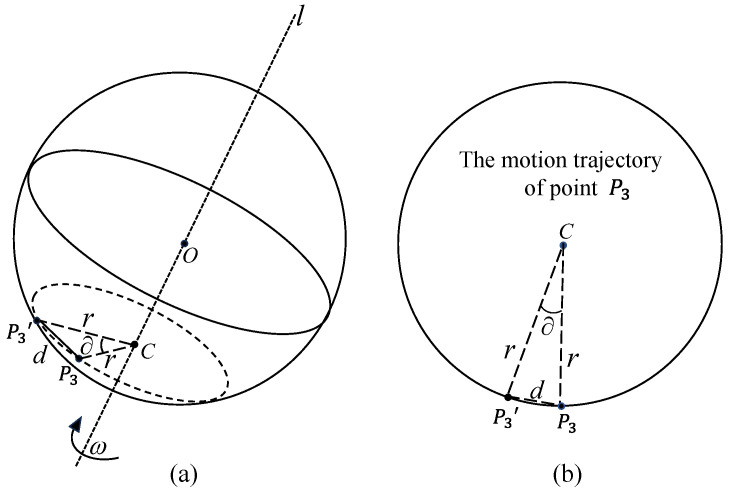
Ball spin angle calculation: (**a**) Schematic diagram of ball spin angle. (**b**) The vertical view of the motion trajectory of point $P_{3}$.

Then find the angular velocity of the spin of the ball. In Figure 4, to solve the spin angular velocity of the marker point $P_{3}$, for example, where ∂ denotes the spin angle of the marker point $P_{3}$, r denotes the radius of the spatial circle ⊙C, and d denotes the distance between the marker point $P_{3}$ and $P_{3}^{\prime }$, the spin angle of the ball in unit time can be expressed as:

(16) $$\partial =2\arcsin(\frac{d}{2r})$$

Let the time interval between the moments before and after the movement of the ball be t, according to Equation (16), the spin angular velocity of the marker point can be found as:

(17) $$\omega =\frac{2\arcsin(\frac{d}{2r})}{t}$$

The distance d can be solved according to the formula for the distance between any two points in space:

(18) $$\left\{\begin{array}{c} d_{P_{1}P_{1}^{\prime }}=\sqrt{{\left(x_{P_{1}}-x_{P_{1}^{\prime }}\right)}^{2}+{\left(y_{P_{1}}-y_{P_{1}^{\prime }}\right)}^{2}+{\left(z_{P_{1}}-z_{P_{1}^{\prime }}\right)}^{2}}\\ d_{P_{2}P_{2}^{\prime }}=\sqrt{{\left(x_{P_{2}}-x_{P_{2}^{\prime }}\right)}^{2}+{\left(y_{P_{2}}-y_{P_{2}^{\prime }}\right)}^{2}+{\left(z_{P_{2}}-z_{P_{2}^{\prime }}\right)}^{2}}\\ d_{P_{3}P_{3}^{\prime }}=\sqrt{{\left(x_{P_{3}}-x_{P_{3}^{\prime }}\right)}^{2}+{\left(y_{P_{3}}-y_{P_{3}^{\prime }}\right)}^{2}+{\left(z_{P_{3}}-z_{P_{3}^{\prime }}\right)}^{2}} \end{array}\right.$$

Substituting Equation (18) into Equation (17), the spin angular velocity at each marker point is solved for:

(19) $$\left\{\begin{array}{c} \omega_{P_{1}}=\frac{2\arcsin(\frac{\sqrt{{\left(x_{P_{1}}-x_{P_{1}^{\prime }}\right)}^{2}+{\left(y_{P_{1}}-y_{P_{1}^{\prime }}\right)}^{2}+{\left(z_{P_{1}}-z_{P_{1}^{\prime }}\right)}^{2}}}{2R_{A}})}{t}\\ \omega_{P_{2}}=\frac{2\arcsin(\frac{\sqrt{{\left(x_{P_{2}}-x_{P_{2}^{\prime }}\right)}^{2}+{\left(y_{P_{2}}-y_{P_{2}^{\prime }}\right)}^{2}+{\left(z_{P_{2}}-z_{P_{2}^{\prime }}\right)}^{2}}}{2R_{B}})}{t}\\ \omega_{P_{3}}=\frac{2\arcsin(\frac{\sqrt{{\left(x_{P_{3}}-x_{P_{3}^{\prime }}\right)}^{2}+{\left(y_{P_{3}}-y_{P_{3}^{\prime }}\right)}^{2}+{\left(z_{P_{3}}-z_{P_{3}^{\prime }}\right)}^{2}}}{2R_{C}})}{t} \end{array}\right.$$

The spin angular velocities of points $P_{1}$, $P_{2}$, and $P_{3}$ are $\omega_{P_{1}}$, $\omega_{P_{2}}$, and $\omega_{P_{3}}$, respectively. For the remaining points $P_{4}$, $P_{5}$, $P_{6}$, …, $P_{n}$, the corresponding spin angular velocities $\omega_{P_{4}}$, $\omega_{P_{5}}$, $\omega_{P_{6}}$, …, $\omega_{P_{n}}$ can be calculated for each point in the same way. Let the spin angular velocity of the ball be ω. In order to reduce the measurement error of the ball spin angular velocity, the average value of the spin angular velocity of each point is taken as the measurement result of the ball spin angular velocity:

(20) $$\omega =\frac{\sum_{n=1}\omega_{P_{n}}}{n}$$

### 2.3. Ball Revolution Speed Measurement Principle

**Figure 5:**
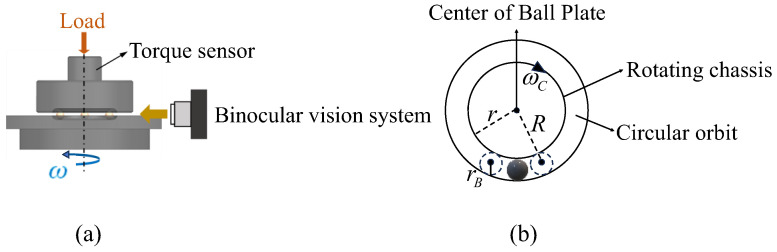
Measurement of ball bearing revolution speed: (**a**) Schematic diagram of ball bearing revolution. (**b**) The top view of the ball bearing revolution.

The time series method is used to measure the revolution speed of the ball as shown in Figure 5. In Figure 5b, r is the radius of the turntable, $r_{B}$ is the radius of the ball, R is the radius of the ball’s revolution, and $\omega_{C}$ is the angular velocity of the turntable. Firstly, record the position of the ball in any image as the initial position and extract the pixel coordinates of that initial position. The shooting time of each image can be clearly given by computer instructions; therefore, the time t when the ball is in its initial position can be recorded. If the time $t_{1}$ for the ball to reach its initial position again after one revolution can be calculated, then the time difference ($t_{1}$ − t) is the period of the ball’s revolution. Since the camera uses discrete imaging, the position of the ball in the image after a cycle of rotation deviates from the initial position, making it impossible to record the time $t_{1}$ directly. To address this problem, select multiple images where the ball appears in the camera’s field of view after one revolution and extract the pixel coordinates of the ball in these images to preliminarily determine the corresponding image when the ball returns to its initial position. Furthermore, select the images with the balls located on the left and right sides of the initial position, and record the corresponding camera shooting time. Finally, a linear equation is set up to solve the equation and to calculate the ball revolution period so that the ball revolution speed can be calculated.

**Figure 6:**
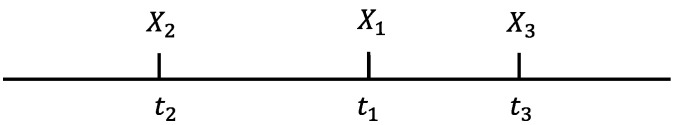
Ball revolution speed measurement principle.

As shown in Figure 6, the time at which the ball first reaches the initial position is noted as t, and the time at which it returns to the initial position again after a cycle of rotation is noted as $t_{1}$. The transverse coordinate of the ball in the pixel coordinate system is labeled as $X_{1}$. The times when the ball is located to the left and to the right of its initial position after a cycle of rotation are labeled as $t_{2}$ and $t_{3}$, respectively. And $t_{2}$ and $t_{3}$ are the corresponding shooting times of the camera, which can be directly displayed by computer commands. The transverse coordinates are labeled as $X_{2}$ and $X_{3}$, respectively.

Then, the lateral coordinates of the ball and the movement time satisfy the following relationship:

(21) $$\frac{X_{3}-X_{1}}{t_{3}-t_{1}}=\frac{X_{1}-X_{2}}{t_{1}-t_{2}}$$

And $t_{1}$ can be obtained:

(22) $$t_{1}=\frac{t_{2}(X_{3}-X_{1})+t_{3}(X_{1}-X_{2})}{X_{3}-X_{2}}$$

The time t at which the ball first reaches its initial position is known; therefore, the ball revolution period is:

(23) $$T=t_{1}-t=\frac{t_{2}(X_{3}-X_{1})+t_{3}(X_{1}-X_{2})}{X_{3}-X_{2}}-t$$

Thus, the ball revolution speed $\omega_{R}$ can be obtained:

(24) $$\omega_{R}=\frac{2\pi }{T}=\frac{2\pi (X_{3}-X_{2})}{t_{2}(X_{3}-X_{1})+t_{3}(X_{1}-X_{2})-t(X_{3}-X_{2})}$$

Then, the sliding velocity $V_{s}$ of the ball can be calculated using the following formula:

(25) $$V_{s}=\omega_{C}r-(\omega r_{B}+\omega_{R}r)$$

## 3. Experimental Measurement of the Proposed Method

**Figure 7:**
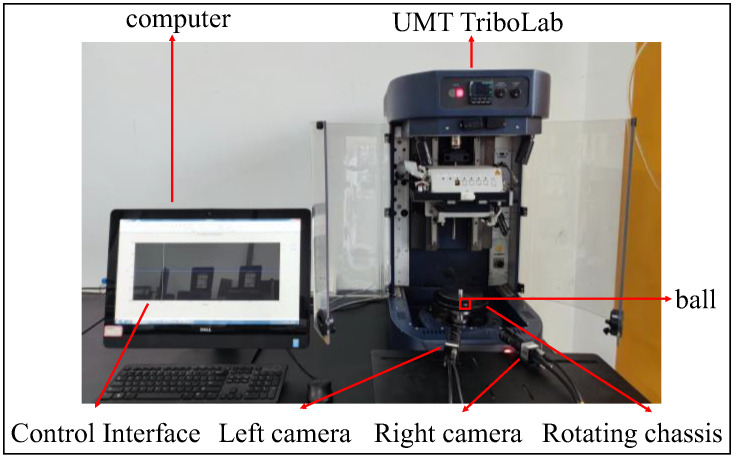
Ball rotational speed measurement platform.

The proposed on-line measurement method of ball rotation vectors was verified on the ball vision measurement platform. As shown in Figure 7, the device includes two black-and-white industrial cameras, a general-purpose mechanical tester, and a computer. The industrial camera model ids UI-3160CP Rev.2.1 has a resolution of 1920 × 1200 pixels and a frame rate of up to 169 FPS (frames per second). The main test stand for ball rotational speed measurement is the Bruker UMT TriboLab. The computer is a Windows 10 operating system with an Intel(R) Core (TM) i5-7400 (The manufacturer is Intel Corporation of Santa Clara, CA, USA) processor. In this experiment, two black-and-white industrial cameras are used to take real-time pictures of the moving balls, and the Bruker Universal Mechanical Tester controls the periodic rotation of the balls at a constant revolution speed. The diameter of the ball used in the experiment is 12.7 mm and the diameter of the rotating chassis is 100 mm.

### 3.1. Experimental Environment Setting

Before using the camera to take pictures of the movement of the ball, the following operations were performed:

(1) Blackening treatment was applied to the edges of the turntable, and interference light in the environment was appropriately blocked to solve the problem that the marker points could not be identified due to the serious reflection on the surface of the metal ball.

(2) A series of marker points are made on the surface of the ball with obvious color differences so that the marker points captured by the camera are clearer. In addition, the configuration of the marks is similar to the intersection of the longitude and latitude lines on the surface of the globe. This ensures that the left and right cameras see enough marker points in the image captured at any given moment.

### 3.2. Experimental Results and Analysis

**Figure 8:**
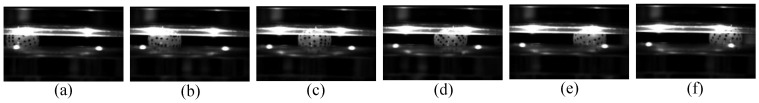
Images taken by the left camera at the moment before the ball movement: (**a**) t = 0 s. (**b**) t = 0.02 s. (**c**) t = 0.04 s. (**d**) t = 0.06 s. (**e**) t = 0.08 s. (**f**) t = 0.10 s.

**Figure 9:**
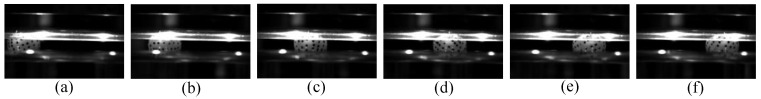
Images taken by the right camera at the moment before the ball movement: (**a**) t = 0 s. (**b**) t = 0.02 s. (**c**) t = 0.04 s. (**d**) t = 0.06 s. (**e**) t = 0.08 s. (**f**) t = 0.10 s.

In order to analyze the motion pattern of the ball over a period of time, several images were taken from the moment the ball entered the field of view to the moment it left the field of view. Use the left and right cameras to synchronously capture images of the ball moving in the raceway as shown in Figure 8 and Figure 9. The moment when the ball first appears completely within the field of view was recorded as 0 and captured a frame rate of 100 FPS for a total of 12 images. Among them, 0 s, 0.02 s, 0.04 s, 0.06 s, 0.08 s, and 0.10 s are the previous moments of the ball movement, and 0.01 s, 0.03 s, 0.05 s, 0.07 s, 0.09 s, and 0.11 s are the subsequent moments of the ball movement. The time interval before and after the ball movement is 0.01 s.

**Figure 10:**
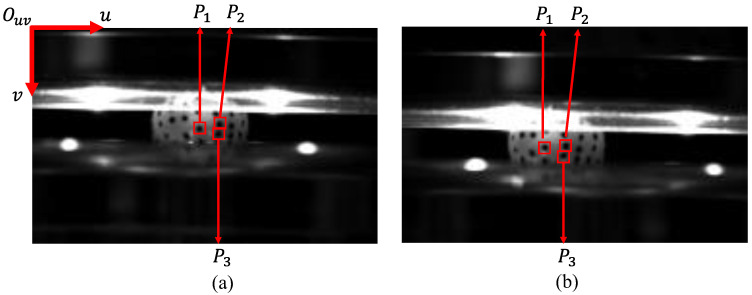
In the pixel coordinate system of the image, the distribution of marker points $P_{1}$, $P_{2}$, and $P_{3}$ in the left and right images: (**a**) Left image. (**b**) Right image.

At t = 0.04 s, the distribution of marker points $P_{1}$, $P_{2}$, and $P_{3}$ in the left and right images are shown in Figure 10. The 2D coordinates of these points in the left and right images were extracted separately, and the results are shown in Table 1.

The rotational speed of the turntable is set to 5 rpm. Calculating the 3D coordinates of these six points based on the pre-calibration parameters of binocular vision [35]. The 3D coordinates of the marker points in the coordinate system of the left camera can be measured at the moment before and the moment after the ball movement. The calculation results are shown in Table 2.

The 3D coordinates of the marker points $P_{1}$, $P_{1}^{\prime }$, $P_{2}$, $P_{2}^{\prime }$, $P_{3}$, $P_{3}^{\prime }$ are known. According to (8), (9), (14), (15) and (18), the Levenberg–Marquardt algorithm was used to optimize and solve the 3D coordinates $A(x_{A},y_{A},z_{A})$, $B(x_{B},y_{B},z_{B})$, $C(x_{C},y_{C},z_{C})$, and $O(x_{o},y_{o},z_{o})$ [36]. The calculation results are shown in Table 3.

**Figure 11:**
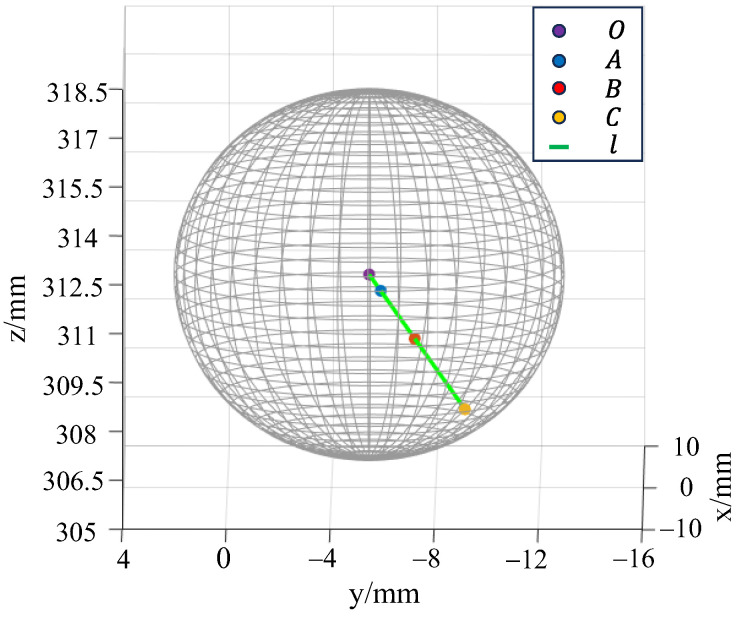
Rotation axis, space circle center, ball center, and the spatial position of the ball.

In order to verify whether the solution of the center coordinates satisfies the three spatial constraints determined in Section 2.2, the rotation axis l, the center of the spatial circle A, B, C, the ball center O, and the spatial position of the ball are displayed, as shown in Figure 11. Obviously, the centers A, B, and C of the three space circles are collinear with the ball center coordinates O at four points, and the actual measurement results conform to the three geometric constraints identified in Section 2.2.

**Figure 12:**
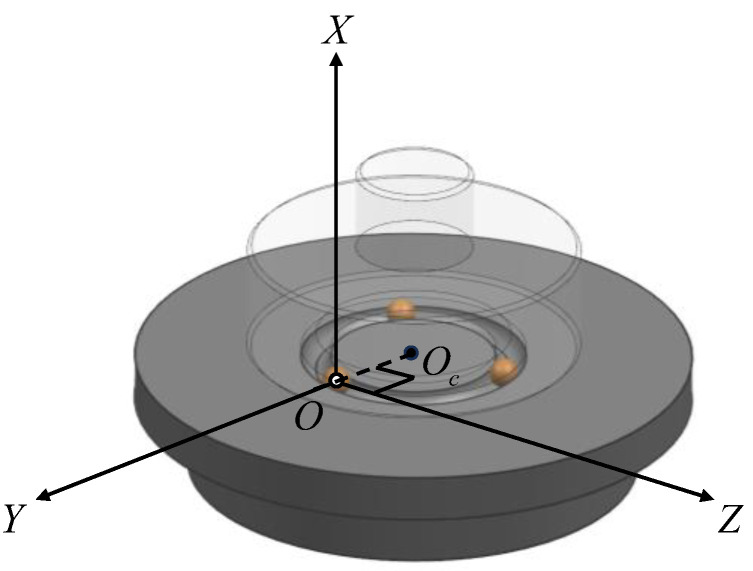
Spherical center coordinate system.

Knowing the 3D coordinates of the marker points $P_{1}$, $P_{1}^{\prime }$, $P_{2}$, $P_{2}^{\prime }$, $P_{3}$, $P_{3}^{\prime }$ and the radii of the spatial circle $R_{A}$, $R_{B}$, and $R_{C}$, according to (23) and (24), the magnitude of the spin angular velocities of the ball can be calculated at 0 s, 0.02 s, 0.04 s, 0.06 s, 0.08 s, and 0.10 s, and the spin angular velocity of the ball is decomposed along the three axis directions of the spherical coordinate system. The spherical coordinate system is based on the origin of the ball center O as shown in Figure 12.

**Figure 13:**
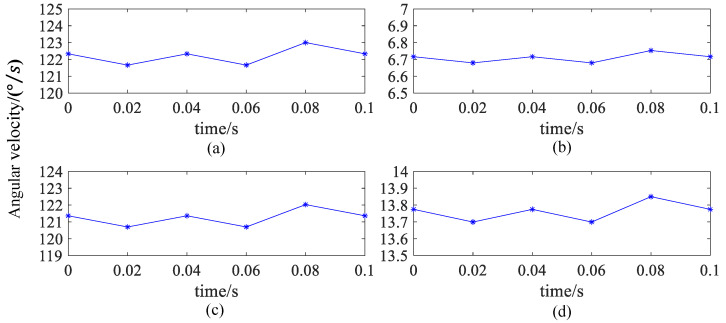
When the chassis speed is 5 rpm, the magnitude of the spin angular velocity of the ball and the decomposition of the spin angular velocity of the ball along the X, *Y*, and *Z* axis: (**a**) Total angular velocity. (**b**) *X*-axis. (**c**) *Y*-axis. (**d**) *Z*-axis.

The *X*-axis is perpendicular to the plane where the turntable is located and faces upwards. The *Z*-axis is tangent to the contact point between the ball and the raceway and points towards the direction of the ball’s revolution speed. The *Y*-axis is perpendicular to the plane where the *X*-axis and *Z*-axis are located and satisfies the right-hand rule. In this way, the spin angular velocities of the ball around the *X*-axis, *Y*-axis, and *Z*-axis can be obtained. When the chassis speed is 5 rpm, the measurement results of the spin angular velocity of the ball bearings are shown in Figure 13.

Figure 13a shows that the spin angular velocity of the ball is almost the same during the motion in 0~0.10 s. The spin angular velocity of the ball shows a consistent increasing or decreasing trend, and the change is relatively stable, with small fluctuations around 122 °/s. Figure 13b–d shows that the spin angular velocities around the *X*, *Y*, and *Z* axis of the spherical coordinate system are 6.7 °/s, 121.0 °/s, and 13.7 °/s, respectively. Moreover, the spin angular velocity presents a relatively consistent variation rule with that shown in Figure 13a. Especially, the spin angular velocity has the largest component around the *Y*-axis in the spherical coordinate system, while the variation rule of the angular velocity is most similar to that in Figure 13a. The spin angular velocity has the smallest component around the *X*-axis in the spherical coordinate system, while the changing trend of the angular velocity is the most stable. The spin angular velocity around the *X*-axis and *Z*-axis is much smaller than the spin angular velocity around the *Y*-axis, which means that the ball moves at its maximum speed in the direction tangential to the chassis and at its minimum speed in the direction perpendicular to the chassis plane. This is consistent with the expected theoretical results.

**Figure 14:**
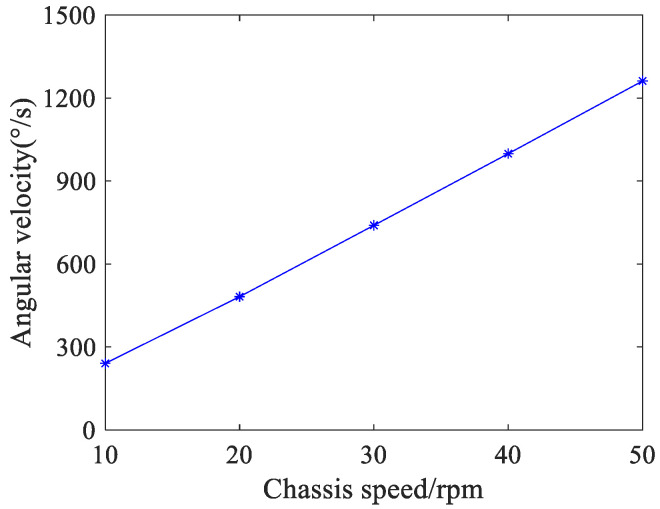
Spin angular velocities of the ball with different chassis speeds.

The measurement results of the spin angular velocities are shown in Figure 14. When the chassis speed is 10 rpm, 20 rpm, 30 rpm, 40 rpm, and 50 rpm respectively, the spin angular velocity of the ball is 241, 482, 740, 1001, and 1262 °/s, respectively. It is obvious that with the increase in the chassis speed multiple, the spin angular velocity increases linearly. Moreover, for every 10 rpm increase in chassis speed, the spin angular velocity increases by about 260 °/s.

**Figure 15:**
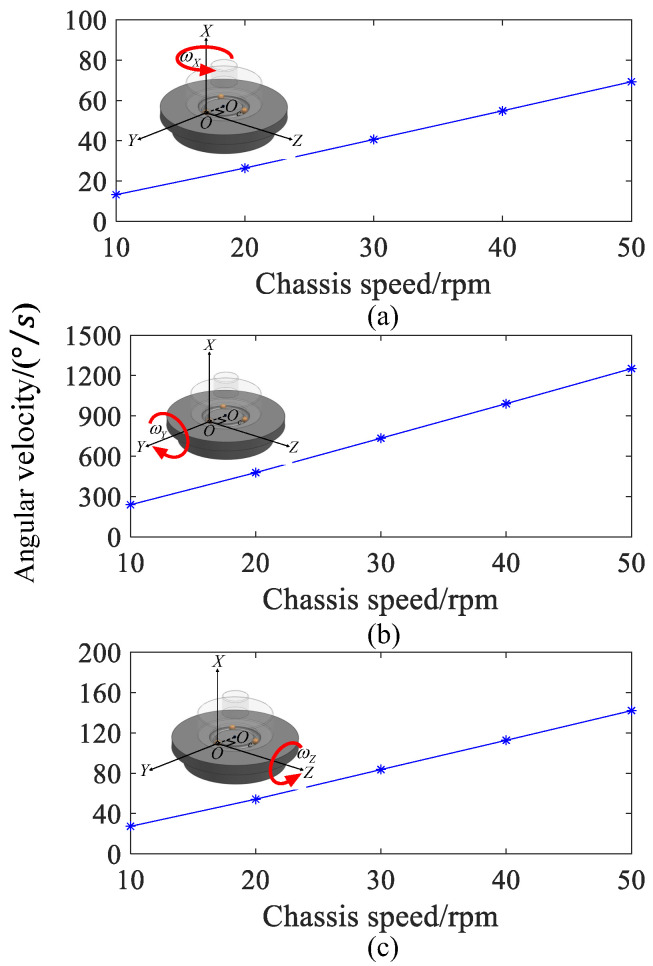
The ball spin angular velocity is decomposed along the axis of the spherical coordinate system: (**a**) *X*-axis. (**b**) *Y*-axis. (**c**) *Z*-axis.

At 0.06 s, with the chassis speed of 10 rpm, 20 rpm, 30 rpm, 40 rpm, and 50 rpm respectively, the spin angular velocity of the ball is decomposed along the three axes directions of the spherical coordinate system to obtain the spin angular velocity of the ball around the *X*, *Y*, and *Z* axes, as shown in Figure 15. As the chassis speed multiplies, the spin angular velocity of the ball around the *X*, *Y*, and *Z* axes all show a linear upward trend. Figure 15a shows that when the chassis speed is 10 rpm, the spin angular velocity of the ball around the *X*-axis is 13.2 °/s; when the chassis speed is 50 rpm, the spin angular velocity of the ball around the *X*-axis is 69.3 °/s. For every 10 rpm increase in chassis speed, the spin angular velocity of the ball around the *X*-axis increases by 14.2 °/s. In addition, the spin angular velocity of the ball around the *X* and Z axes is much smaller than the spin angular velocity around the *Y* axis, which is consistent with the expected theoretical results. Figure 15b,c shows that the spin angular velocity of the ball around the *Y* and *Z* axes have the same variation rule as shown in Figure 15a. Furthermore, when the chassis speed increases by 10 rpm, the spin angular velocity around the *Y* and *Z* axes increases by 257.9 °/s and 29.3 °/s, respectively.

**Figure 16:**
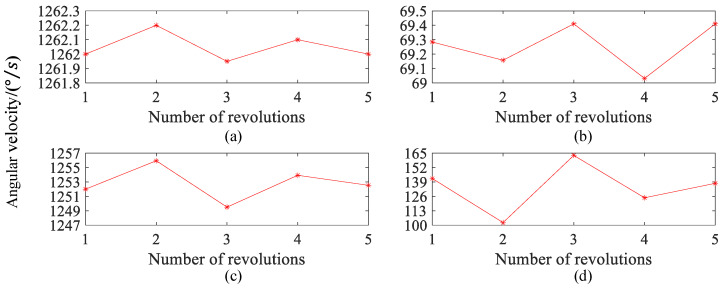
The spin angular velocity and the decomposition of spin angular velocity around the *X*, *Y*, and *Z* axes of the ball at different turns: (**a**) Total angular velocity. (**b**) *X*-axis. (**c**) *Y*-axis. (**d**) *Z*-axis.

When the chassis speed is set to 50 rpm, calculate the spin angular velocity of the ball after stable motion for the 1–5th turn and t = 0.06 s. The measurement results are shown in Figure 16. Obviously, the spin angular velocity of the ball stabilizes around 1262 °/s at 0.06 s per revolution, indicating that the total spin angular velocity of the ball is almost constant. The spin angular velocity in the second circle is the highest at 1262.20 °/s, while, in the third circle, the spin angular velocity is the lowest, at 1261.95 °/s. The maximum difference in angular velocity and measurement error are 0.25 °/s and 0.18%, respectively.

As shown in Figure 16b–d, the spin angular velocities of the ball around the *X*, *Y*, and *Z* axes are different at different turns. In addition, the spin angular velocity variation of the ball around the *X*-axis is the smallest, with a maximum angular velocity difference of 0.38 °/s. The spin angular velocity variation around the *Z*-axis is the largest, with a maximum angular velocity difference of 60.65 °/s. The maximum angular velocity difference around the *Y*-axis is 6.44 °/s, and the variation pattern of the angular velocity component of the ball around the *Y*-axis is consistent with the overall angular velocity. It is worth noting that whenever the spin angular velocity component of the ball around the *Y*-axis increases, the angular velocity components around the *X* and *Z* axes both decrease and vice versa. Analyzing the reasons for the above phenomenon, the total spin angular velocity of the ball is almost constant; however, as the ball continues to move, the direction of its spin’s angular velocity constantly changes. This results in a constant change in the angular velocity component of the ball around each coordinate axis, which further confirms the change in the direction of spin of the ball during its motion.

**Figure 17:**
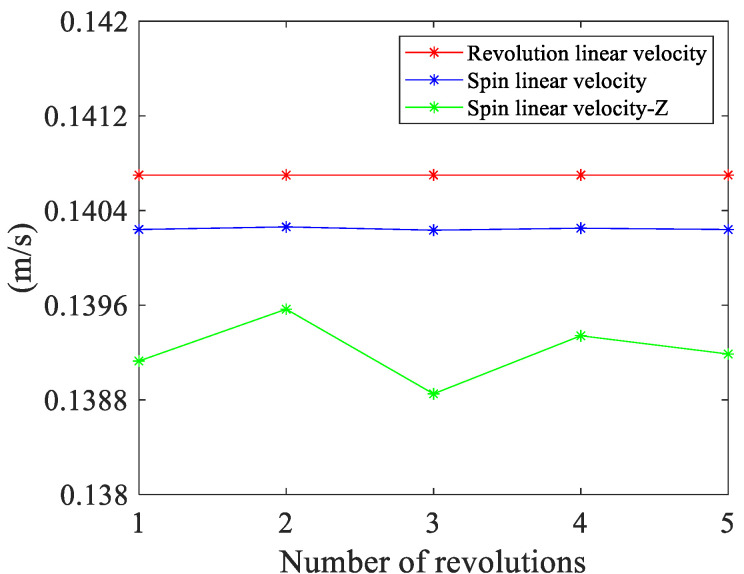
The revolution linear velocity, spin linear velocity, and spin linear velocity along the *Z*-axis in the spherical coordinate system at different turns.

When the chassis speed is 50 rpm, the measured revolution speed of the ball is 26.8 rpm, which is 160.8 °/s. Calculate the revolution linear velocity, total spin linear velocity and spin linear velocity along the *Z*-axis for the 1~5 turns after stable ball motion. The measurement results are shown in Figure 17. The red line represents the revolution linear velocity of the ball, the blue line represents the spin linear velocity of the ball, and the green line represents the spin linear velocity along the tangent direction of motion. It shows that the revolution linear velocity of the ball is a fixed value of 140.7 mm/s and the spin linear velocity of the ball is also stable at 140.2 mm/s. Moreover, the maximum difference and measurement error of the ball spin linear velocity is 0.028 mm/s and 0.19%, respectively, indicating that the measurement accuracy of the proposed method can reflect micrometer-level errors and achieve good measurement results.

On the other hand, the spin linear velocity of the ball along the tangent direction of motion fluctuates slightly around 139.2 mm/s. Considering the reason for this difference, due to the limited frame rate of the camera used, it is impossible to guarantee that the position of the ball in the image is completely consistent after each revolution. The above part has proven that when the ball moves to a different position, the spin direction of the ball changes, and the scalar of the spin linear velocity of the ball is constant. This causes the magnitude of the spin linear velocity, which is decomposed into the tangent direction, which is also changing. What is more, the spin linear velocity of the ball along the tangent direction of motion is slightly smaller than the revolution linear velocity, while the difference between them is 1.5 mm/s.

## 4. Discussion

**Figure 18:**
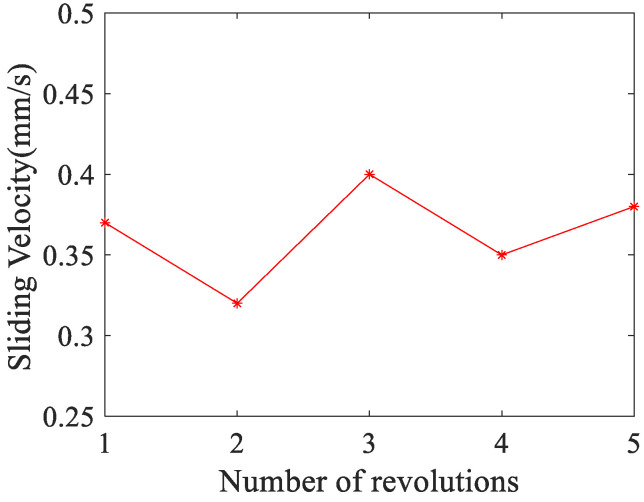
The sliding velocity of the ball at different turns.

Analyze the sliding phenomenon of the ball during movement. When the turntable speed is set to 50 rpm, the revolution speed of the ball is 26.8 rpm. At this point, the revolution linear velocity of the ball is a fixed value of 140.7 mm/s, and the spin linear velocity of the ball is also stable at 140.2 mm/s. According to Equation (25), the sliding velocity of the ball is calculated, and the result is shown in Figure 18.

According to Equation (25), as the spin angular velocity of the ball increases, the sliding velocity of the ball will decrease, and vice versa. It can be inferred from this that the variation law of ball sliding velocity is completely opposite to the variation law of ball spin angular velocity. Combining Figure 16a and Figure 18 for analysis, the variation law of the two are indeed opposite. This further proves the effectiveness of this method for measuring the sliding velocity of bearing balls, which can achieve monitoring of the operating status of bearings. Given that the speed of the ball is 26.8 revolutions per minute, which means the rotation period of the ball is 2.24 s, the sliding distance between the ball and the chassis after a cycle of rotation is 0.67 mm.

At present, we have achieved the measurement of the rotational vectors of the bearing ball on the Bruker friction and wear equipment. In practical applications, we have carried out measurements of bearing balls in real operating conditions using control moment gyroscope (CMG) test system (CMG test system built according to the real operating environment). As long as the partial area of the ball is visible in the section plane, the high precision spin angular velocity and linear velocity can be measured according to the short rolling time of the ball. Then, the sliding behavior and running state of the ball are analyzed and monitored, respectively.

In practical applications, the selection of lubricating oil will also affect the sliding of the bearing balls. Different lubricating oil has different viscosity, and the viscosity of lubricating oil is one of several factors that affect the traction force of ball movement and thus the sliding behavior of bearing balls. If the viscosity of the lubricating oil is too small, the tractive force will decrease, which will make the contact interface oil film easy to damage; consequently, both the direct contact slide of the interface and the friction will increase. In general, viscosity increases traction and decreases sliding. However, when the viscosity is too large, the wear of the interface and lubricant will also be greatly increased. In addition, temperature is also one of the factors affecting the sliding behavior of the bearing ball. The ball constantly reciprocates in the bearing, and there is a certain friction between the ball and the raceway, and the contact interface temperature rises. This results in a decrease in the viscosity of the lubricating oil, which in turn increases the relative slide between the ball and the raceway. This paper mainly focused on the establishment of the ball spin motion model and the measurement of the ball rotational vectors under real rotation conditions, proving the correctness and applicability of the method. Therefore, the sliding characteristics under different lubricating oil, temperature, humidity, and pressure conditions were not further analyzed. However, these factors will become the main work of our next research.

## 5. Conclusions

We develop a novel method for measuring the spin angular velocity of bearing balls based on the three-dimensional coordinates of feature points, and the binocular vision technology is first applied to the measurement of the rotation vectors of the bearing balls. Through the binocular vision measurement system and the ball spin motion model constructed, the motion vectors of the ball were measured effectively and accurately, and the sliding behavior of the ball was analyzed. The experimental results show that the measurement error range of spin angular velocity is [0.21 °/s, 0.25 °/s], and the measurement error range of linear velocity is [0.023 mm/s, 0.028 mm/s]. Specifically, by measuring several groups of bearing balls at different chassis speeds, we control the measurement error within ±0.2%, which indicates that the measurement system has good measurement accuracy. The proposed method shows that the real-time measurement of rotational vectors such as spin angular velocity of bearing balls can be realized by the measurement of three-dimensional coordinates of binocular vision feature points. This is of great significance for the analysis of the sliding behavior of bearing balls in the mechanical field and the observation of the operating status of bearings.

## Figures and Tables

**Table 1:** Two-dimensional coordinates of marker points in the left and right image.

Point	2D Coordinates in Left Image	2D Coordinates in Right Image
$P_{1}$	(929.75, 566)	(796.25, 674)
$P_{2}$	(1043.75, 540)	(911.75, 666.5)
$P_{3}$	(1036.25, 593)	(899.75, 719)

**Table 2:** Three-dimensional coordinates of marker points.

Point	X Coordinate/mm	Y Coordinate/mm	Z Coordinate/mm
$P_{1}$	−5.46	−7.18	307.62
$P_{2}$	−2.14	−8.90	307.17
$P_{3}$	2.94	−9.58	307.91
$P_{1}^{\prime }$	−1.86	−6.22	306.74
$P_{2}^{\prime }$	0.10	−8.18	306.96
$P_{3}^{\prime }$	4.12	−9.02	308.22

**Table 3:** Center coordinates and radius of the ball.

Point	Three-Dimensional Coordinate	Radius
A	(−1.24, −5.90, 312.51)	6.30
B	(−2.56, −7.21, 311.64)	5.85
C	(−4.50, −9.16, 308.56)	4.48
O	(−0.79, −5.45, 312.81)	6.37

## Data Availability

Data are contained within the article.

## References

[1] Han Q., Chu F. (2015). Nonlinear dynamic model for skidding behavior of angular contact ball bearings. J. Sound Vib..

[2] Gao S., Han Q., Pennacchi P., Chatterton S., Chu F. (2023). Dynamic, thermal, and vibrational analysis of ball bearings with over-skidding behavior. Friction.

[3] Yu Y., He Y., Karimi H.R., Gelman L., Cetin A.E. (2024). A two-stage importance-aware subgraph convolutional network based on multi-source sensors for cross-domain fault diagnosis. Neural Netw..

[4] Oktaviana L., Tong V.C., Hong S.W. (2019). Skidding analysis of angular contact ball bearing subjected to radial load and angular misalignment. J. Mech. Sci. Technol..

[5] Xu T., Xu G., Zhang Q., Hua C., Tan H., Zhang S., Luo A. (2013). A preload analytical method for ball bearings utilising bearing skidding criterion. Tribol. Int..

[6] Gupta P.K. (1979). Dynamics of rolling-element bearings part IV: Ball bearing results. J. Tribol..

[7] Chen W., Ma Z., Gao L., Li X., Pan J. (2012). Quasi-static analysis of thrust-loaded angular contact ball bearings part I: Theoretical formulation. Chin. J. Mech. Eng..

[8] Östensen J., Åström H., Höglund E. (1995). Analysis of a grease-lubricated roller bearing under arctic conditions. Proc. Inst. Mech. Eng. Part J J. Eng. Tribol..

[9] Imado K. (2000). Fundamental study of hall element method to detect ball motion in ball bearings. Tribol. Trans..

[10] Selvaraj A., Marappan R. (2011). Experimental analysis of factors influencing the cage slip in cylindrical roller bearing. Int. J. Adv. Manuf. Technol..

[11] Wang W.Z., Hu L., Zhang S.G., Zhao Z.Q., Ai S. (2014). Modeling angular contact ball bearing without raceway control hypothesis. Mech. Mach. Theory.

[12] Ye M., Liang J., Li L., Qian B., Ren M., Zhang M., Lu W., Zong Y. (2021). Full-field motion and deformation measurement of high speed rotation based on temporal phase-locking and 3D-DIC. Opt. Lasers Eng..

[13] Bao R., Duan F., Fu X., Yu Z., Liu W., Guo G. (2023). Frequency-scanning interferometry for axial clearance of rotating machinery based on speed synchronization and extended Kalman filter. Opt. Lasers Eng..

[14] Wang L., Bi S., Li H., Gu Y., Zhai C. (2020). Fast initial value estimation in digital image correlation for large rotation measurement. Opt. Lasers Eng..

[15] Jia R., Xue J., Lu W., Song Z., Xu Z., Lu S. (2024). A Coupled Calibration Method for Dual Cameras-Projector System with Sub-Pixel Accuracy Feature Extraction. Sensors.

[16] Yu C., Ji F., Xue J., Wang Y. (2019). Adaptive binocular fringe dynamic projection method for high dynamic range measurement. Sensors.

[17] Xu J., Zheng S., Sun K., Song P. (2023). Research and application of contactless measurement of transformer winding tilt angle based on machine vision. Sensors.

[18] Furuno S., Kobayashi K., Okubo T., Kurihara Y. (2009). A study on spin-rate measurement using a uniquely marked moving ball. Proceedings of the 2009 ICCAS-SICE.

[19] Liao Y.H., Wang L., Yan Y. (2022). Instantaneous rotational speed measurement of wind turbine blades using a marker-tracking method. Proceedings of the 2022 IEEE International Instrumentation and Measurement Technology Conference (I2MTC).

[20] Wang Y., Wang L., Yan Y. (2017). Rotational speed measurement through digital imaging and image processing. Proceedings of the 2017 IEEE International Instrumentation and Measurement Technology Conference (I2MTC).

[21] Wang T., Wang L., Yan Y., Zhang S. (2018). Rotational speed measurement using a low-cost imaging device and image processing algorithms. Proceedings of the 2018 IEEE International Instrumentation and Measurement Technology Conference (I2MTC).

[22] Wang T., Yan Y., Wang L., Hu Y. (2018). Rotational speed measurement through image similarity evaluation and spectral analysis. IEEE Access.

[23] Wang T., Yan Y., Wang L., Hu Y., Zhang S. (2019). Instantaneous rotational speed measurement using image correlation and periodicity determination algorithms. IEEE Trans. Instrum. Meas..

[24] Wang S., Xu Y., Zheng Y., Zhu M., Yao H., Xiao Z. (2018). Tracking a golf ball with high-speed stereo vision system. IEEE Trans. Instrum. Meas..

[25] Jung J., Park H., Kang S., Lee S., Hahn M. (2010). Measurement of initial motion of a flying golf ball with multi-exposure images for screen-golf. IEEE Trans. Consum. Electron..

[26] Seo S.W., Kim M., Kim Y. (2018). Optical and acoustic sensor-based 3D ball motion estimation for ball sport simulators. Sensors.

[27] Zhang Z., Xu D., Tan M. (2010). Visual measurement and prediction of ball trajectory for table tennis robot. IEEE Trans. Instrum. Meas..

[28] Xie Z., Yang C. (2024). Binocular Visual Measurement Method Based on Feature Matching. Sensors.

[29] Zhang Y., Xiong R., Zhao Y., Wang J. (2015). Real-time spin estimation of ping-pong ball using its natural brand. IEEE Trans. Instrum. Meas..

[30] Li W., Jin J., Li X., Li B. (2010). Method of rotation angle measurement in machine vision based on calibration pattern with spot array. Appl. Opt..

[31] Zhong J., Zhong S., Zhang Q., Peng Z., Liu S., Yu Y. (2018). Vision-based measurement system for instantaneous rotational speed monitoring using linearly varying-density fringe pattern. IEEE Trans. Instrum. Meas..

[32] Kim H., Yamakawa Y., Senoo T., Ishikawa M. (2016). Visual encoder: Robust and precise measurement method of rotation angle via high-speed RGB vision. Opt. Express.

[33] Guo J., Zhu C., Lu S., Zhang D., Zhang C. (2016). Vision-based measurement for rotational speed by improving Lucas–Kanade template tracking algorithm. Appl. Opt..

[34] Ma S., Yin Y., Chao B. (2023). A real-time coupling model of bearing-rotor system based on semi-flexible body element. Int. J. Mech. Sci..

[35] Lu X., Xue J., Zhang Q. (2020). High camera calibration method based on true coordinate computation of circle center. Chin. J. Lasers.

[36] Moré J.J. (2006). The Levenberg-Marquardt algorithm: Implementation and theory. Proceedings of the Numerical Analysis: Proceedings of the Biennial Conference, Dundee, Scotland, 28 June–1 July 1977.

